# Perspectives of San Juan healthcare practitioners on the detection deficit in oral premalignant and early cancers in Puerto Rico: a qualitative research study

**DOI:** 10.1186/1471-2458-11-391

**Published:** 2011-05-26

**Authors:** Douglas E Morse, Carmen M Vélez Vega, Walter J Psoter, Himilce Vélez, Carmen J Buxó, Linda S Baek, Augusto Elias, Melba Sánchez Ayendez

**Affiliations:** 1 Department of Epidemiology & Health Promotion, New York University College of Dentistry, 250 Park Avenue South, Room 633, MC: 9479, New York, NY, 10003-1402, USA; 2 Department of Social Sciences, Graduate School of Public Health, University of Puerto Rico Medical Sciences Campus, San Juan, 00936, PR; 3 Public Health Program, Ponce School of Medicine, Ponce, 00716, PR; 4 Office of the Assistant Dean of Research (A141E), School of Dental Medicine, University of Puerto Rico Medical Sciences Campus, GPO Box 365067, San Juan, 00936-5067 PR; 5 Formerly, Department of Human Development, Graduate School of Public Health, University of Puerto Rico Medical Sciences Campus, San Juan, PR

## Abstract

**Background:**

In Puerto Rico, relative to the United States, a disparity exists in detecting oral precancers and early cancers. To identify factors leading to the deficit in early detection, we obtained the perspectives of San Juan healthcare practitioners whose practice could be involved in the detection of such oral lesions.

**Methods:**

Key informant (KI) interviews were conducted with ten clinicians practicing in or around San Juan, Puerto Rico. We then triangulated our KI interview findings with other data sources, including recent literature on oral cancer detection from various geographic areas, current curricula at the University of Puerto Rico Schools of Medicine and Dental Medicine, as well as local health insurance regulations.

**Results:**

Key informant-identified factors that likely contribute to the detection deficit include: many practitioners are deficient in knowledge regarding oral cancer and precancer; oral cancer screening examinations are limited regarding which patients receive them and the elements included. In Puerto Rico, specialists generally perform oral biopsies, and patient referral can be delayed by various factors, including government-subsidized health insurance, often referred to as Reforma. Reforma-based issues include often inadequate clinician knowledge regarding Reforma requirements/provisions, diagnostic delays related to Reforma bureaucracy, and among primary physicians, a perceived financial disincentive in referring Reforma patients.

**Conclusions:**

Addressing these issues may be useful in reducing the deficit in detecting oral precancers and early oral cancer in Puerto Rico.

## Background

Worldwide in 2008, there were an estimated 263,000 new cases of and 127,000 deaths attributed to cancer of the lip and oral cavity (ICD-10 C00-C08) [1]. Oral cancer incidence rates for Puerto Rico are among the highest in the Western Hemisphere, particularly among males for whom age-adjusted rates (world, all ages included) were an estimated 8.0/100,000 in 2008 [2]. Recently, Suarez, et al. reported that during the period 1998-2002, age-standardized incidence rates for oral and pharyngeal cancer (C00-C14) increased in Puerto Rico for both males and females [3].

Most cancers of the oral cavity and lip are squamous cell carcinomas, which often arise from red and/or white precursor lesions presenting on the surface mucosa [4-7]. It is generally agreed that such lesions should be biopsied and evaluated histopathologically; however, oral precancers and early cancers are often asymptomatic and can go undetected in the absence of a careful oral examination [4,5,8,9]. In the case of premalignant lesions, a biopsy and histopathologic diagnosis provide information as to how closely a lesion should be followed and whether treatment should be initiated to prevent malignant transformation. Early detection of *in situ *and early invasive oral cancers is important because 5-year survival rates are notably higher for persons diagnosed with early, relative to late, stage disease [10,11]. Further, oral cancer patients with early-stage disease often require less radical cancer-directed treatment, suffer fewer associated sequellae, and have a better health-related quality of life than cases diagnosed with late-stage disease [12,13].

In previous reports we identified a significant disparity in the biopsying of a) potentially precancerous oral lesions and b) *in situ *oral cancers in Puerto Rico relative to the United States [14,15]. Our findings suggest that Puerto Rico residents with intraoral lesions suspicious for oral cancer or precancer are most likely to be biopsied only after developing an invasive cancer and therefore, are not receiving the benefits of early detection.

In order to meaningfully address shortfalls in the detection of oral precancer and early-stage cancer in Puerto Rico, it is important to first consider the potentially diverse perspectives of healthcare practitioners on the Island in terms of their knowledge, attitudes/beliefs and practices related to oral precancer and early cancer diagnosis [14,15]. Toward that aim, key informant (KI) interviews were conducted with a spectrum of healthcare professionals whose practices, in or around San Juan, Puerto Rico, could be involved in the detection of oral precancer and early cancer. We then triangulated our KI interview findings with other data sources, including recent literature on oral cancer detection from various geographic areas, current curricula at the University of Puerto Rico (UPR) Schools of Medicine and Dental Medicine, as well as local health insurance regulations.

## Methods

The study was approved by the Institutional Review Boards of the UPR Medical Sciences Campus and New York University.

The purposive selection of key informants was centered on the need to obtain multiple perspectives on the research domains and to cover the range of healthcare practitioners who could be involved in the detection of oral precancer and early cancers. Healthcare practitioners selected for participation in the KI interviews included general dentists (GD), primary care physicians (PCP), otolaryngologists (ENT), oral surgeons (OS), and dental assistants/hygienists (DAH). Our sample was limited to practitioners who practiced in or around the San Juan Metropolitan Area, i.e., Old San Juan, Santurce, Hato Rey, Río Piedras, Caimito, Cupey, Guaynabo, Bayamón, Cataño, Carolina, and Trujillo Alto.

Recruitment of KIs was carried out by one of the investigators (AEB) in consultation with other members of the research team as well as with local dental, medical, and dental hygiene societies. Initially, male and female practitioners who met the inclusion criteria and represented a range in terms of years in practice, healthcare role, and practice profiles were identified. From among the identified clinicians, potential KIs were then purposively selected to provide both the predetermined number of participants and multiple perspectives on the topics of interest. Potential KIs were contacted in a systematic fashion by AEB, invited to participate, and for those who agreed, an appointment scheduled. The process continued until the required number of practitioners, by type, was achieved. Each participant received a $250 honorarium for the time required to complete the interview.

### Interview Guide

KI interviews utilize a semi-structured interview guide which, while having a specific organization and flow, permits flexibility to obtain open-ended responses and to follow-up with probes and additional questions on issues initiated by respondents. The interview guide was created with input from a panel of eight study personnel whose expertise and professional backgrounds included oral healthcare, oral cancer, public health, cultural anthropology, and qualitative research methodology. The guide was developed in English, translated into Spanish, and corroborated for linguistic, cultural, and social intricacies pertinent to Puerto Rico. The guide was then reviewed and modifications suggested by a JAPICO Community Board (Junta Asesora para la Prevención e Investigación de Cancer Oral de Puerto Rico, or Advisory Council for the Prevention and Research into Oral Cancer in Puerto Rico), which was established at the UPR Medical Sciences Campus as part of an oral cancer center grant [16]. The interview guide was further refined in terms of language and comprehension by pretesting the instrument with three healthcare professionals (OS, ENT, DAH) [17,18]. The final guide consisted of sequence questions designed to elicit detailed information, while associated probing questions allowed informants to elaborate on each of their responses. The guide included questions relating to professional and continuing education, the impact of health insurance on health-seeking and care, the performance of oral cancer examinations and oral biopsies as well as other factors that could be related to the observed deficit in early oral cancer diagnosis.

### Key Informant Interview Process

All health professionals were interviewed in their offices with the exception of one individual who came to the UPR Medical Sciences Campus for that purpose. Interviews were conducted in Spanish on a one-to-one basis by a single interviewer, a cultural anthropologist with experience in qualitative public health research (MS). The process began with an explanation of the research objectives, intended uses of the information, and assurances of confidentiality. All participants completed the consent process by signing a consent form that included permission to record the interview.

### Data Analysis

All KI interview audio files were transcribed, and the transcriptions verified. The product text was then sent to the primary analyst, who completed the initial analysis, including the coding of lists and categories. The process of analyzing the interview texts began using an inductive process in which major themes were identified. Themes and supporting quotes were then reviewed and classified into inclusive categories. These categories were integrated using codes developed by the main analyst from the interview instrument. The resulting list of codes was then used to analyze the interview texts by a deductive method. Texts that represented the codes were then quoted in order to sustain or validate findings. The process was organized using qualitative analysis (Altas.ti) software. The categories and supporting texts were further analyzed; coinciding and complimentary categories were further integrated into a list of codes.

Codes produced in the initial inductive analysis were compared to the list of codes from the interview guide and then organized into broad categories responding to the study objectives and research questions. The process of analysis, including quote selection, was performed first individually by each of the three qualitative researchers and subsequently by a consensus process with input from members of the entire research team.

## Results

Saturation during the analysis process is indicative of having a more than adequate study sample size as it suggests that more interviews will not produce new information. We experienced saturation in the text analysis as we reached the transcript for the fourth KI interview, and as we completed analysis of the tenth and final KI interview, no new codes or categories were generated.

Ten of the eleven practitioners approached to take part in the interviews agreed to participate - two primary care physicians, two general dentists, two ENT specialists, two oral surgeons, and two dental auxillaries, i.e., one dental hygienist and one registered expanded-functions dental assistant. Each participant practiced in or around the San Juan metro area; there were six male and four female KIs. All interviews were conducted during the period February 1-13, 2008, and each participant answered all questions. Interview length varied from 45 minutes to approximately two hours.

### Professional Background and Practice Characteristics

All informants had practiced exclusively in Puerto Rico. We purposively selected four practitioners who had practiced for 9-12 years and six who had practiced for over 16 years. Each KI treated primarily adults and reported having previously cared for patients covered by government health insurance. At the time of the interview, six KIs treated primarily patients with government insurance, and four served mainly patients with private insurance coverage.

The current form of government-subsidized health insurance (GSHI) is termed Puerto Rico Health Reform (Reforma de Salud de Puerto Rico), or simply Reforma [19] and is utilized largely, but not exclusively, by persons of lower socioeconomic status (SES). At the time of the interview, most KIs continued to care for patients from lower SES groups; however, two practitioners no longer treated patients with Reforma-based GSHI.

### Reasons for Delays in the Diagnosis of Oral Diseases

#### Socioeconomic Status

All KIs felt that socioeconomic status had an impact on the duration that patients wait prior to seeking care, with many lower SES patients delaying significant oral health needs for months or years. Patient factors identified as contributing to delays in health seeking among those of lower SES included limited financial resources and lack of education.

I believe that the lower the educational and socioeconomic level [of the population], the more we will have a negative response [not seeking oral health care] in relation to what we are talking about [oral cancer] (OS).

#### Government-Subsidized Health Insurance (GSHI)

While related to lower SES status, most KIs felt that GSHI was also detrimental to the provision of adequate healthcare by making it more difficult and time consuming for patients to receive needed attention, with the delay largely attributed to "red tape," i.e., "bureaucratic complexities." The topic of GSHI in relation to diagnostic delay is addressed further under the heading of 'Biopsies.'

#### Knowledge related to Early Detection

Most participants stated that a major barrier to the diagnosis of oral precancer and early cancer is a lack of knowledge regarding early signs and symptoms on the part of *both *the lay population and healthcare professionals.

Patients are not reached by trained professionals with the knowledge or the intuition to suspect a precancerous lesion; evidently [due to] lack of education, lack of knowledge (OS).

Because oral premalignant and early cancers are not routinely observed by most general practitioners, one general dentist felt that periodic training on the clinical presentation of oral precancerous and cancerous lesions is needed in order to keep the material fresh in the minds of practitioners.

Perhaps lack of knowledge of a lesion as a [precancerous/cancerous] lesion; I believe that since there is not more frequent training on oral cancer lesions, if we do not see and do not know, the less you see, the less you know (GD).

#### Professional Training

Most participants could identify some previous professional training related to oral cancer prevention, identification, and treatment, primarily as a component within a course that dealt with a more comprehensive subject such as oral diagnosis or oncology.

They did offer something, yes, but not a class on oral cancer or the diagnosis of oral cancer itself. They offered oral diagnosis, but not oral cancer (GD).

Two participants (OS, ENT) stressed the importance of formal professional education in oral cancer detection. One ENT expressed the need for more comprehensive training in which the theoretical, conceptual and practical aspects of cancer prevention and identification were included in the professional curriculum.

On a more cynical note, one KI felt that some clinicians preferred not to be educated on the detection of oral cancer and precancer.

In many occasions, I believe the lack of knowledge is not a lack of having been provided the information during training, but instead that the professional does not care to be educated in this respect (OS).

#### Continuing Education (CE)

Only three KIs (OS, DAH, GD) reported having attended a CE course that directly addressed the topic of oral cancer during the preceding three years. Reasons given for not attending an oral cancer CE course during the past three years included the lack of offerings and unfavorable reviews from colleagues who had attended the meetings.

I have not attended, because when I have received information about these courses, they do not attract my attention, because it is my understanding that the speakers of these courses will not offer a high quality conference (OS).

#### Oral Cancer Examinations and Early Detection

A number of KIs felt that the current status of early oral cancer detection in Puerto Rico was problematic.

Honestly, very poor [referring to early oral cancer detection in Puerto Rico] because realistically, it [oral cancer] is not discovered as much because people [health practitioners] do not perform oral exams on patients. They do not open their mouths. Sometimes people arrive with something they have had for months, and no one [checks the mouth] (PCP).

Most participants stated that the healthcare professional with the greatest responsibility for providing an oral cancer examination is the general dentist although some felt that all health professionals should perform opportunistic oral cancer examinations.

It could be any health professional, be it a physician, generalist, or specialist related to oral health (OS).

Many of the KIs stated that they had at least some previous training in conducting oral cancer examinations; however, others (GD, both PSPs) had not.

I do not recall having been taught how to perform an oral exam in any moment (PCP)

Concern was voiced as to the quality of oral cancer examinations being conducted.

I think they [clinicians] are far from the reality of performing a good oral soft tissue exam. I don't know if it is due to fear or lack of training, but they are far from the reality of searching for, or going in search of, [oral abnormalities] as part of the exam (OS).

Among those KIs who reported having received at least some training in performing an oral cancer examination, many reported conducting visual-tactile examinations (VTE). Based upon the interviews, however, it became clear that a consistent definition was lacking as to what constitutes a visual-tactile examination, and only two KIs (OS, ENT) described a VTE as including visualization and palpation of the oral cavity, head, and neck. The majority of KIs emphasized the importance of visualizing the oral structures during oral cancer examinations, with less importance placed on the tactile component.

With the caveat that the term "visual-tactile examination" was self-defined by and not consistent across all practitioners, three KIs (OS, both ENTs) reported that they conducted a VTE on all adult patients while four (OS, PCP, both GDs) reported they performed an oral cancer examination only on high-risk patients or when there was a specific patient concern about a possible abnormality. Two KIs (DAH, PCP) reported they did not perform VTEs on any patients, and one KI (DAH) stated that only visual examinations were performed at her present practice. For those KIs who reported they would perform a complete VTE only when they considered the patient to be at high risk, most agreed that the factors used to determine risk were age (40+), gender (male), a history of smoking and/or heavy drinking, low socioeconomic status, and poor hygiene.

I perform them [VTE] mainly on high-risk patients, drinkers and smokers, but I do not perform them routinely on all patients (GD).

For most participants, risk assessment was judged to be a more important element than a tactile examination.

One KI (ENT) felt that the oral cancer CE courses he attended made the assumption that everyone had a common knowledge as to what constitutes and how to perform a thorough VTE.

It was assumed that everyone knew how to perform a tactile examination with both hands (ENT).

Conversely, one KI (GD) reported a change in practice toward performing visual and tactile examinations after attending a hands-on CE course that recommended a thorough tactile examination and provided the opportunity to learn how to perform it.

One participant recommended that CE credits on the topic of oral cancer be mandatory for all healthcare professionals.

It [oral cancer CE] should be as compulsory as once were courses for CPR and breastfeeding (GD).

#### Biopsies

When asked about who should perform oral biopsies, the general opinion was that such procedures are the domain of surgeons, i.e., ENTs, oral surgeons.

The oral surgeons and ENTs reported that they routinely perform oral biopsies when they detect a suspicious lesion.

I can detect a precancerous or cancerous lesion, and I perform a biopsy immediately during the initial visit (OS).

On the other hand, general dentists and primary care physicians reported that they refer patients who require an oral biopsy to specialists.

If I see a suspicious lesion, I inform the patient that there is a rare lesion, and I send the patient to the oral surgeon, because I do not perform biopsies (GD).

When referring a patient with a suspicious lesion, practitioners did so with the knowledge that the referral could result in a delay in obtaining both the biopsy and subsequent diagnosis. For example, some practitioners acknowledged that delays in diagnosis may arise due to issues related to coordinating the referral. Such difficulties were most often described in terms of government-subsidized health insurance; however, it was stated that the referral process could also be hindered by other forms of insurance that require preauthorization.

If the patient has private insurance that covers services, this person will seek treatment directly from the specialist. This person will go directly to the ENT or oral surgeon or periodontist; you know, he will call and get an appointment, has insurance pay for services and receives a diagnosis. Patients from health systems such as government insurance and HMOs have to go through a process of authorizations (ENT).

On a related note, it was also stated that referrals can be delayed by a medical office administrator who does not move the referral documentation ahead in a timely manner.

There was considerable confusion on the part of some informants as to GSHI guidelines relating to oral biopsy referrals, and two informants (PCP and GD) felt that perceived problems with GSHI often result from a lack of knowledge on the part of clinicians regarding insurance coverage and how to use it to the advantage of the patient.

The capitated nature of GSHI applies to many healthcare practitioners in Puerto Rico, but not dentists. Because referrals made under a capitated system can be regarded by some practitioners as financial liabilities to be avoided, a few KIs were concerned that monetary considerations can arise that influence when and when not to refer a patient.

Reforma gives great responsibility to primary physicians in making these decisions - limiting or not, his work capital [capitation payments]. These are really the conflicts that can be created. But in reality, without biopsies and diagnosis, options for treatment cannot be provided. Yes, they [the patients] are affected (PSP).

Further, some practitioners discussed the fact that patients themselves often delay their biopsy appointment due to fear of the biopsy, fear of a diagnosis of cancer or other major disease, or fear of associated costs. For example, when GSHI payments are lower than the usual and customary rate, patient out-of-pocket expenses may create an obstacle to receiving a timely biopsy.

[The general dentists] are going to refer and even if the oral surgeon accepts Reforma, somehow it ends up costing [the patient] $100 for the consult (GD).

In order to avoid delays in obtaining a biopsy and diagnosis, three KIs (GD, OS, DAH) felt that general dentists, and perhaps primary care physicians, should receive more training in performing oral biopsies.

Recently-graduated health professionals do not know how to recognize cancer; second, they do not know how to perform a biopsy. In other words, for me, having received training thirty-something years ago gave us an excuse, but at a moment when cancer is so important and [there is] so much concern and leagues against cancer and battles against cancer, it is a concern that a youngster [young health professional] does not know how to perform a biopsy (GD).

## Discussion

Recent findings provide strong evidence that in Puerto Rico, oral lesions suspicious for precancer or early cancer are most likely to be biopsied only after developing an invasive cancer [14,15]. Such findings are important because delays in oral cancer diagnosis can have substantial consequences in terms of the treatment required, quality of life, and long-term survival.

In order to obtain insight into the factors that contribute to the apparent delay in biopsying and diagnosing lesions suspicious for early oral cancer and precancer, qualitative data were obtained using KI interviews with healthcare practitioners whose practice could be involved in the detection of oral precancer and early cancers. By design, the group of KIs covered a range of healthcare practitioners who practiced in the Greater San Juan Metropolitan Area and could offer diverse perspectives on the research topics of interest. During these interviews, the key informants identified three broad areas they believed account for a substantial portion of the apparent delays, i.e., the healthcare-seeking public, healthcare practitioners, and the insurance system. The current paper focuses largely on the latter two areas; subsequent papers, based on focus groups comprised of lay individuals, will explore perspectives of the healthcare-seeking public.

### Reasons for Diagnostic Delays

#### Socioeconomic Status of the Health-seeking Public (SES)

Most of the health practitioners felt that the delay in oral cancer diagnosis was inversely related to level of patient socioeconomic status. However, while there is good evidence that low, relative to high, socioeconomic status is associated with an increased risk of oral cancer [20], the impact of SES on diagnostic delay has received little attention and is currently poorly understood for oral [21] and many other cancers [22]. Whether or not SES independently impacts oral cancer diagnostic delay in Puerto Rico, lower socioeconomic status is clearly related to GSHI, another factor identified by participants as an important predictor of delays in the detection of oral precancers and early cancers.

#### Government-Subsidized Health Insurance (GSHI)

GSHI, the expanded Medicaid program for Puerto Rico, or Reforma, is a managed healthcare program that was enacted in 1993 and phased in by geographical area beginning in 1994 [23,24]. In general, Reforma covers Puerto Rico residents who are underinsured earning up to 200% of the local poverty level; public sector employees, veterans and their families; persons eligible for Medicaid, and Medicare recipients without complete coverage [25,26]. Reforma currently covers an estimated 40% of Island residents [23]. Although healthcare, as provided through Reforma, is largely a capitated program, the provision of dental services by dentists and dental specialists is on a fee-for-service basis [24]. Our KI interviews revealed that many practitioners are not clear on various Reforma guidelines and requirements, with much of the confusion centering on biopsy referrals.

### Knowledge related to oral precancer and early cancer detection

#### Professional Training

Some KIs reported that deficits in the detection of oral precancers and early cancers were due, at least in part, to a limited background knowledge of oral cancer and its signs and symptoms among many clinicians on the Island, a finding echoed in previous studies involving physicians and dentists in other geographic areas [27-29]. The consistency in findings across studies, in addition to the perceived frontline status of dentists and other healthcare practitioners in the identification of oral precancer and early cancer, argue that both dental and medical students receive a strong background in oral cancer signs, symptoms, and clinical presentation during their professional training.

All participating KIs completed their formal education at least nine years prior to being interviewed and therefore, with the exception of the specialist participants, may have had less oral cancer training than more recent dental and medical school graduates. This is notable in that previous studies conducted on the United States mainland suggest that time since graduation from dental or medical school is inversely related to oral cancer knowledge [30-32].

Most dentists and primary care physicians currently practicing in Puerto Rico received their professional health training on the Island. A recent review of the four-year curriculum at the UPR School of Dental Medicine revealed that there are currently ten courses in the undergraduate dental curriculum that include information on oral cancer and precancer, e.g., oral diagnosis and treatment planning, oral pathology (C. Krespo, personal communication). At the UPR Medical School, courses that cover oral cancer are also integrated into the curriculum. While it is beyond the scope of the current study to evaluate the effectiveness of medical and dental school curricula in Puerto Rico as regards oral cancer, previous studies on the United States mainland have recommended that medical and dental schools in general increase their training on the topic of oral cancer [31,33-36]. Possible programmatic additions to the medical and dental curricula in Puerto Rico, including those of applicable residencies, could include requirements for demonstrating skills in a) knowing risk factors for the disease, b) identifying oral lesions suspicious for precancer and early cancer, and c) performing oral cancer examinations [37,38]. Licensure examinations could also be expanded to include testing of the same skills.

#### Continuing Education (CE)

Evolving information on oral cancer risk factors, examination techniques, and changing trends in cancer incidence and mortality highlights the need for clinicians to periodically update their knowledge on oral cancer-related topics. Nevertheless, based upon responses of health practitioners involved in our interviews, oral cancer CE has not been a priority for the majority of participants. Similarly, studies conducted on the United States mainland have revealed that a sizable proportion of US-based dentists had either never attended a CE course on oral cancer or had done so years ago [35,39,40]. The recency of oral cancer CE attendance is important because studies have found it to be positively associated with measures [37,39] and perceptions of current oral cancer knowledge [36] and possibly, the thoroughness of the oral cancer examination [39]. While oral cancer CE received mixed reviews among the study KIs, it is notable that one participant who had attended such CE reported changing clinical practice by including a tactile component in the oral cancer examination. It is also notable that one respondent called for oral cancer CE to be required for relicensure of all Puerto Rico healthcare professionals, a recommendation voiced in a previous US report [37].

#### Oral Cancer Examinations

Studies conducted in the US have found that dentists are more likely than physicians to report performing opportunistic oral cancer examinations [31,41], and as in previous investigations [27,42], there was broad agreement among the study participants that dentists have the major responsibility for providing such examinations. It is noteworthy however, that a subset of the KIs felt that such examinations should be routinely provided by other health professionals as well.

Most, but not all, of the KIs reported at least some training in performing oral cancer screenings, and as with previous reports, lack of training was a contributing factor for failing to conduct oral cancer examinations [27,38,40]. To highlight the importance of such examinations, it has been suggested that dental schools require their students to demonstrate competency in performing oral cancer examinations [38], and such a proficiency may have utility at the Puerto Rico Schools of Medicine and Dental Medicine. As stated above, it also appears prudent that medical and dental applicants for licensure in Puerto Rico be required to demonstrate proficiency in performing an oral cancer examination.

In keeping with previous reports [38-40,43,44], the participants indicated that their current conduct of opportunistic oral cancer screenings is often limited in terms of the elements included in the examination and the patients who receive them. Although visual oral examinations have reported utility in increasing the detection of early-stage oral cancers [45] and in reducing the risk of oral cancer mortality in tobacco or alcohol users [46], it has long been advocated that oral examinations screening for cancer and precancer should incorporate both a visual and tactile component, which includes palpation of cervical lymph nodes and the major salivary glands [47-53]. However, many participating KIs limited their oral cancer examinations to visual inspections. Further, although it has been advocated that all adult patients, and not just those at elevated risk, be opportunistically screened for oral cancer and precancer, at least in the dental setting [48], many KIs indicated that they routinely provide an oral cancer examination only for persons with characteristics that place them at "high-risk."

The observation that many Puerto Rico practitioners are limiting their opportunistic oral cancer screening examinations, both in terms of which patients receive an examination and which, if any, sites are palpated, is consistent with reports from previous investigations in other geographic regions [38-40]. Of note, in one US-based report, dentists felt more confident than family physicians in their training to perform a visual oral cancer examination while physicians were more confident than dentists in palpating cervical lymph nodes [54].

Apart from training, variation in oral cancer examination practices may result, at least in part, from the lack of definitive consensus-generated guidelines that specifically state which patients should be examined, which elements should be included in a routine oral cancer examination, and how often such examinations should be performed in asymptomatic patients [38,49,55]. Further, statements reading, for example, "there is insufficient evidence to support or refute the use of a visual examination as a method of screening for oral cancer" [56] can easily be *misinterpreted *as stating that opportunistic visual oral cancer examinations have no value. A recent report from an expert panel convened by the American Dental Association to evaluate current evidence regarding the possible risks and benefits of oral cancer screening addressed some of the above issues by recommending that "clinicians remain alert for signs of potential malignant lesions or early-stage cancers while performing routine visual and tactile examinations in all dental patients, but particularly those who use tobacco or consume alcohol heavily" [8].

#### Biopsy

The current gold standard for diagnosing oral cancer and precancer is a scalpel or punch biopsy followed by a histopathologic diagnosis [4,5,8,9,57]. Based upon our KI interviews, it appears that in Puerto Rico, most oral biopsies are performed by oral and maxillofacial surgeons and ENTs, with other dental and medical practitioners generally referring to those specialists. Surveys in various other regions of the world have also found that most patients requiring an oral biopsy are referred to specialists [58-61].

Some participating KIs believed that general dentists and perhaps primary care physicians should receive additional training in performing biopsies, a practice that could reduce professional-, system-, and/or patient-based delays in the diagnosis of oral abnormalities. Reports from areas outside Puerto Rico have reflected the same sentiment, particularly with regard to biopsying apparently benign lesions. Others, however, have voiced concerns that lack of expertise on the part of some general practitioners may result in inappropriate or inadequate biopsies as well as a disruption in the clinical presentation of suspicious lesions, thereby leading to delays in the definitive diagnosis of oral precancers and cancers [58]. Further, some general practitioners may excise a lesion but lack sufficient knowledge in terms of appropriate patient management when a histopathologic diagnosis is received [59]. These concerns argue against many general dentists and primary care physicians biopsying lesions suspicious for oral precancer/cancer and highlight the potential benefits in having such procedures performed by practitioners with advanced training whenever possible. If the current standard of care for lesion biopsy among general dentists and primary care physicians in Puerto Rico is referral to a specialist, it is imperative that practitioners be familiar not only with requirements put forth by insurance entities, including GHSI, but also the importance of making an appropriate referral.

In the United States and for many cancers, health insurance status is a significant predictor of stage at diagnosis, with a higher likelihood of advanced stage disease for persons with either Medicaid or no health insurance coverage relative to those with private insurance [62,63]. In our interviews, Government-subsidized health insurance (Reforma) was consistently cited as problematic in the timely provision of oral biopsies although other health insurance plans requiring preauthorizations prior to biopsy were also identified. The situation with regard to Reforma is exacerbated by a) a lack of information on the part of many patients and practitioners regarding the basic provisions of GSHI, b) a perceived financial disincentive on the part of primary care physicians for referring Reforma patients to specialists for oral biopsies, and c) diagnostic delays related to sometimes time-consuming bureaucratic GSHI authorizations. These and other issues associated with healthcare under GSHI have received detailed attention in previous comprehensive reports [23,24].

### Study Limitations

The findings of the current qualitative investigation should be considered in light of study limitations. We elected to include a spectrum of healthcare practitioners who, given their type of practice, could be involved in the detection of oral precancer and early cancer. We also chose to limit our focus to clinicians who practiced in the Greater San Juan Metropolitan Area where the majority of oral health services are concentrated in Puerto Rico. Had we included a greater number of healthcare specialists under each of the practice type headings or interviewed healthcare professionals practicing in other areas of Puerto Rico, we may have obtained additional perspectives on the observed deficit in oral precancer and early cancer identification. Nevertheless, those professionals who did participate were consistent in their viewpoints across the spectrum of practitioner type, and saturation was reached quickly.

We did not ask the study participants about high risk human papillomavirus (HPV), a relatively recently recognized risk factor for primarily squamous cell carcinomas of the oropharynx [64], nor did any of the key informants bring up the topic. It is noteworthy that compared to persons diagnosed with an HPV-negative head and neck cancer, individuals who have an HPV-positive head and neck cancer are more likely to be nondrinkers, nonsmokers, younger at the time of diagnosis and as likely to be female as male [65,66]. The disparate profiles of persons with HPV-positive versus those with HPV-negative head and neck tumors have the potential to redefine how clinicians classify their patients in terms of "high risk" and how they examine them for oral and oropharyngeal cancer.

## Conclusions

Based upon our interviews with clinicians practicing in San Juan and consistent with other data sources, a number of practitioner-based factors were identified as likely contributing to the deficit in oral precancer and early cancer diagnosis on the island of Puerto Rico, as follows:

- Many healthcare professionals, including general dentists, have a limited background knowledge of oral cancer, which can arise from decrements in professional training, a lack of exposure to oral cancer continuing education, and a disinterest on the part of some healthcare practitioners.

- Many practitioners limit their opportunistic oral cancer screening examinations, in terms of which, if any, patients receive them and which elements are included in the examination.

- Oral biopsies are most often performed by specialists, and patient referrals can be delayed by a number of factors. The most frequently mentioned issues centered on government-subsidized health insurance, and identified concerns focused on a) a lack of practitioner knowledge regarding basic requirements and provisions of GSHI, b) PCP-perceived financial disincentives associated with the referral of Reforma patients for oral biopsies, and c) diagnostic delays related to GSHI "red tape."

With the caveat that clinicians practicing in other areas of Puerto Rico may have contributed additional explanations for the observed deficit in oral precancer and early cancer identification, our KI interviews provide insight into the knowledge, attitudes/beliefs and practices of healthcare practitioners whose clinical activities can result in the early detection of such lesions. The implementation of strategies to address the above issues may be useful in reducing the deficit in detecting oral precancers and early oral cancers in Puerto Rico.

## Competing interests

The authors declare that they have no competing interests.

## Authors' contributions section

DEM was the study Principal Investigator, directed the project and participated in all aspects of the study design, field guide development, NYU IRB approvals, interpretation of study results, and primary authorship of the manuscript. CMV played a major role in the study design and development of the interview guide, served as the primary analyst, and was a major contributor in summarizing the study findings. WJP played a major role in designing the study, developing the interview guide, and interpreting results of the study analysis. HV played a major role in the study design and development of the interview guide, served as one of the secondary analysts, and was a major contributor to summarizing the study findings. CJB played an active role in development of the interview guide, oversaw UPR IRB submissions, was instrumental in coordinating the Key Informant interviews, oversaw transcription of the interviews and was a major contributor in the interpretation of study findings. LSB played an active role in the compilation of study documentation, interpretation of study findings, and the writing of the manuscript. AE played an active role in development of the interview guide, was instrumental in identifying and recruiting healthcare practitioners to participate in the Key Informant interviews, identified current curricula related to oral cancer at the University of Puerto Rico Medical Sciences Campus, and was a major contributor to interpreting the study findings. MS played a major role in the study design and development of the interview guide, led the qualitative research group (CMV, HV), conducted all Key Informant interviews, served as a secondary analyst, and was a major contributor to summarizing the study findings. All authors read and approved the final manuscript.

## Pre-publication history

The pre-publication history for this paper can be accessed here:

http://www.biomedcentral.com/1471-2458/11/391/prepub
